# Sulphur doped carbon dots enhance photodynamic therapy via PI3K/Akt signalling pathway

**DOI:** 10.1111/cpr.12821

**Published:** 2020-05-04

**Authors:** Yanjing Li, Shihong Wu, Junjiang Zhang, Ronghui Zhou, Xiaoxiao Cai

**Affiliations:** ^1^ State Key Laboratory of Oral Diseases National Clinical Research Center for Oral Diseases West China Hospital of Stomatology Sichuan University Chengdu China; ^2^ Analytical & Testing Center Sichuan University Chengdu China; ^3^ Department of Prosthodontics Tianjin Medical University Tianjin China

**Keywords:** carbon dots, cell apoptosis, inhibitor, photodynamic therapy, PI3K/Akt

## Abstract

**Objectives:**

Photodynamic therapy (PDT) is a promising approach for cancer treatment, and the underlying signalling pathway changes has been carried out for studying the PDT mechanisms, but is majorly limited to organic photosensitizers (PSs). For the emerging nano‐PSs typically possessing higher ^1^O_2_ quantum yield, few mechanistic studies were carried out, which limited their further applications in clinical therapeutics. PI3K/Akt signalling pathway, a most frequently activated signalling network in cancers, could promote cancer cell survival, but was seldom reported in previous PDT studies mediated by nano‐PSs.

**Materials and Methods:**

Sulphur doped carbon dots (S‐CDs) was prepared via a hydrothermal synthetic route and was characterized by transmission electron microscopy, X‐ray photoelectron spectroscopy and so on. CCK‐8 assay and Annexin V/PI staining were performed to demonstrate the death of cancer cells, Western blot, RT‐PCR and immunofluorescence were employed to explore the underlying mechanism, and variation of PI3K/Akt and other signalling pathways was detected by Western blot.

**Results:**

S‐CDs was successfully synthesized, and it was much more efficient compared with classic organic PSs. S‐CDs could induce cancer cell death through mitochondria mediated cell apoptosis with the imbalance of Bcl‐2 family proteins and caspase cascade via several signalling pathways. Low concentration of S‐CDs could effectively inhibit PI3K/Akt pathway and promote p38/JNK pathway, on one way inhibiting cancer cell survival and on the other way promoting cell apoptosis.

**Conclusions:**

Herein, we found that S‐CDs acted as an inhibitor of the PI3K/Akt pathway for efficient cancer cell killing, thus yielding in a higher PDT performance over the existing photosensitizers.

## 1 INTRODUCTION

Photodynamic therapy (PDT) shows important benefits in minimally invasive or even noninvasive, low toxicity, repeatability, good selectivity and widespread applicability.^1^, ^2^ Irradiation causes the photosensitizers (PSs) to transfer energy to molecular oxygen to generate reactive oxygen species (ROS), especially yield singlet oxygen (^1^O_2_), which subsequently oxidizes biomolecules in the photosensitized tissue, inducing cell death and tumour elimination.^3^, ^4^ Based on previous studies, cell apoptosis is reported the most majority mode of cell death in organic PSs‐based PDT, which is induced by the change of apoptosis‐related biomolecules such as calcium, cytochrome *C* (Cyto *C*) and signalling pathways such as mitogen activated protein kinases (MAPKs).^5^, ^6^, ^7^, ^8^ However, it is worth noting that PDT mediated cell apoptosis could be regulated by other signalling networks in cancers, such as protein kinase B, which could promote cancer cell survival.^9^, ^10^ Thus, it is expected that inhibition of these pathway in PDT can boost further cell death besides p38/JNK pathway.

Phosphatidylinositol‐3‐kinases/protein‐serine‐threonine kinase (PI3K/Akt), a serine/threonine protein kinase, was important in the regulation of key cellular functions such as growth and survival. PI3K/Akt was reported to be one of the most frequently activated signalling networks in human cancers, and the pathway aberrations have been identified in up to 40% of all tumour types.^11^ Akt, activated by PI3K, phosphorylates and inhibits the pro‐apoptotic Bcl‐2 family members, resulting in cell survival and tumoregenesis.^12^, ^13^ Consequently, PI3K/Akt has become an attractive target in anticancer therapy, and it will be of far‐reaching significance if the efficient PS could inhibit the activation of this pathway. Numerous inhibitors targeting the pathway at all levels are now in clinical development, including but not limited to perifosine, Afuresertib (GSK2110183) and AZD5363 (Astrazeneca).^12^ Therefore, searching an efficient PS, which could act as PI3K/Akt inhibitor, will be of great significance for the clinical development of nano‐PSs.

Recently, as organic PSs was limited from poor water solubility and relatively low ROS generation efficiency, nano‐photosensitive agents are widely developed due to the excellent characteristics such as adjustable excitation and emission wavelength, strong anti‐photobleaching ability, small size, low toxicity and good biocompatibility.^14^, ^15^, ^16^, ^17^ Herein, by using the high efficiency nano‐PS, sulphur doped carbon dots (S‐CDs), we for the first time indicated that S‐CDs could instigate potent cancer cells apoptosis as a PI3K/Akt inhibitor, which may lead to the high efficiency for PDT. Glioblastoma multiforme is a malignant primary type of aggressive brain cancer with high proliferation and metastasis rates, and we found that S‐CDs‐PDT could efficiently induce apoptosis of glioblastoma cells (U87‐MG) and inhibit PI3K/Akt signalling pathway. Another cancer cell line A375 (melanoma cell) was employed to further demonstrate the inhibition of PI3K/Akt during S‐CDs‐PDT, and the results showed efficient inhibition of PI3K/Akt and cancer cell survival. By detecting the changes of biomolecules in these cancer cells, including apoptotic factors, apoptotic proteins and signalling pathway proteins, the negative regulator effect of the S‐CDs to the PI3K/Akt pathway was demonstrated.

## 2 MATERIALS AND METHODS

### 2.1 Synthesis and characterization of S‐CDs

The S‐CDs was prepared via a hydrothermal synthetic route. In brief, PT2 as precursor was first synthesized by steps of nucleophilic addition, suzuki reaction and oxidative polymerization from 4‐bromobenzyl bromide.^18^ Then, according to Ge et al,^8^ 6 mg PT2 was dispersed in 8 mL 0.5 mmol/L NaOH solution. After treated the mixture ultrasonically for 30 minutes, it was transferred into an autoclave (20 mL) and heated at 170°C for 24 hours. Then, the mixture was cooled down to room temperature, and S‐CDs were purified through filtering using 0.22‐mm membranes and further dialysed against distilled water. All the instrumental information used for characterizations were given in Table S1.

### 2.2 In vitro cytotoxicity assay

U87‐MG or A375 cells were incubated with different concentration of S‐CDs, PT2 or chlorin e6 (Ce6) at 37°C in 5% CO_2_ for 12 hours, and both PT2 and Ce6 were explored as control. One plate was used for exploring the cytotoxicity of the photosensitizer without irradiation. The other plate was irradiated using ultraviolet light‐emitting diode (LED) (S‐CDs and PT2, λ = 420 nm, Ce6, λ = 660 nm) with an intensity of 25 J/cm for 5 min after taking away the photosensitive agents and exchanging for fresh medium. Then the cell viability was determined by cell counting kit‐8 (CCK‐8). In detail, cells after treatment were incubated with CCK‐8 diluent for 1 hour, and then measured the optical densities (OD) at 450 nm. The experiments were performed 3 times.

Cell apoptosis was determined by Annexin V‐fluorescein isothiocyanate (FITC) and propidium iodide (PI) staining kit. Briefly, after incubation with 0.1 μmol/L photosensitizers for 12 hours, 2 × 10^6^ cells treated with/without irradiation (25 J/cm) were collected in phosphate buffered saline (PBS). After washing with PBS for twice, cells were re‐suspended in 500 μL binding buffer (1×) and stained with 5 μL Annexin V‐FITC and 5 μL PI for 15 minutes in the dark. Finally, the assay was performed by flow cytometry.

### 2.3 Colocalization assay

U87‐MG cells loaded with S‐CDs in confocal dishes were washed with PBS, and then incubated with Mito‐tracker green (a mitochondria probe) or Lyso‐tracker green (a lysosome probe). After incubation at 37°C in 5% CO_2_ for 30 minutes, samples were washed with PBS and observed by confocal laser scanning microscopy. Mito‐tracker green and Lyso‐tracker green was excited at 488 nm, and the emission was collected from 505 to 535 nm.

### 2.4 Western blotting analysis

Cancer cells treated with or without photosensitizers and irradiation were washed with ice‐cold PBS for 3 times to remove the residual medium. Then cells were lysed in lysis buffer to harvest total proteins, and then detected by Nanodrop to measure the protein concentrations. The protein samples were separated on 6%, 10% and 12% (v/v) SDS polyacrylamide gels and transferred into polyvinylidene difluoride (PVDF) membranes. After blocking with 5% bovine serum albumin or dehydrated milk, the membranes were incubated with primary antibodies as following, anti‐Bax (abcam), anti‐Bcl‐2 (abcam), anti‐Caspase‐3 (abcam), anti‐Cyto *C* [EPR1327] (abcam), anti‐PI3K p85α [EPR18702] (abcam), anti‐PI3K p110β [EPR5515(2)] (abcam), anti‐NF‐κB [E379] (abcam), anti‐IκB [E130] (abcam), anti‐JNK1 + JNK2 + JNK3 [EPR16797‐211] (abcam), anti‐JNK1 + JNK2 + JNK3 (phospho T183 + T183 + T221) [EPR5693] (abcam), anti‐Akt [40D4] (CST), anti‐Akt (phospho Ser473) [D9W9U] (CST), anti‐p38 [D13E1] (CST), anti‐p38 (Thr180/Tyr182) [D3F9] (CST) and anti‐GAPDH [14C10] (CST). The housekeeper gene glyceraldehyde‐3‐phosphate dehydrogenase (GAPDH) was employed as an internal control. Then the blots were incubated with secondary antibodies (anti‐rabbit or anti‐mouse, respectively). Finally, chemiluminescence was developed by ECL reagents.

### 2.5 Immunofluorescence

Cancer cells in confocal dish of the two different groups, namely, treated with PDT (PDT group) or without treatment (control group), were fixed and permeabilized. The samples were then blocked by 1% goat serum and cultured with primary antibody solution (anti‐Bax, anti‐Bcl‐2, anti‐Caspase‐3 rabbit polyclonal antibody). Next, cells were incubated with labelled secondary antibody and the nuclei and cytoskeleton were stained with DAPI and phalloidin, respectively. Finally, the samples were observed by confocal laser scanning microscopy (CLSM).

### 2.6 Detection of ROS generation

Cancer cells in confocal dish of the four different groups, control group, incubation with S‐CDs (S‐CDs group), irradiation without S‐CDs (light group) and irradiation after PDT (PDT group), were washed with PBS for twice. All the samples were stained with DCFH‐DA, a ROS probe, and the nuclei were stained with Hoechst33342. The fluorescence of DCFH‐DA was observed by fluorescent inverted microscope immediately.

### 2.7 Detection of free calcium in cells

To detect the level of free Ca^2+^ in cells, cells in four groups, control group, incubation with S‐CDs (S‐CDs group), irradiation without S‐CDs (light group) and irradiation after PDT (PDT group), were incubated with Fluo‐4 AM, a calcium probe. Hoechst33342 was employed to stain the nuclei. The green fluorescence of Fluo‐4 AM was observed by fluorescent inverted microscope immediately.

### 2.8 Quantitative PCR

Total RNA of cancer cells treated with or without PDT were collected, isolated and purified via Trizol extraction method. After being melted in the RNase‐free water, total mRNA was quantified by spectrophotometer. All the mRNA samples were reverse transcribed into cDNA using a synthesis kit. The expression of target mRNAs (Table S2) in each treatment group as normalized to GAPDH was evaluated. The quantitative PCR (qPCR) was performed using SYBR® Green I PCR master mix and an ABI 7300 thermal cycler.

### 2.9 Statistical analysis

Statistical analyses were performed using student's *t*‐test via SPSS 16.0. There was markedly differential in statistics when the values of *P* < .05.

## 3 RESULTS

### 3.1 Synthesis and characters of sulphur doped carbon dots (S‐CDs)

**Figure 1:**
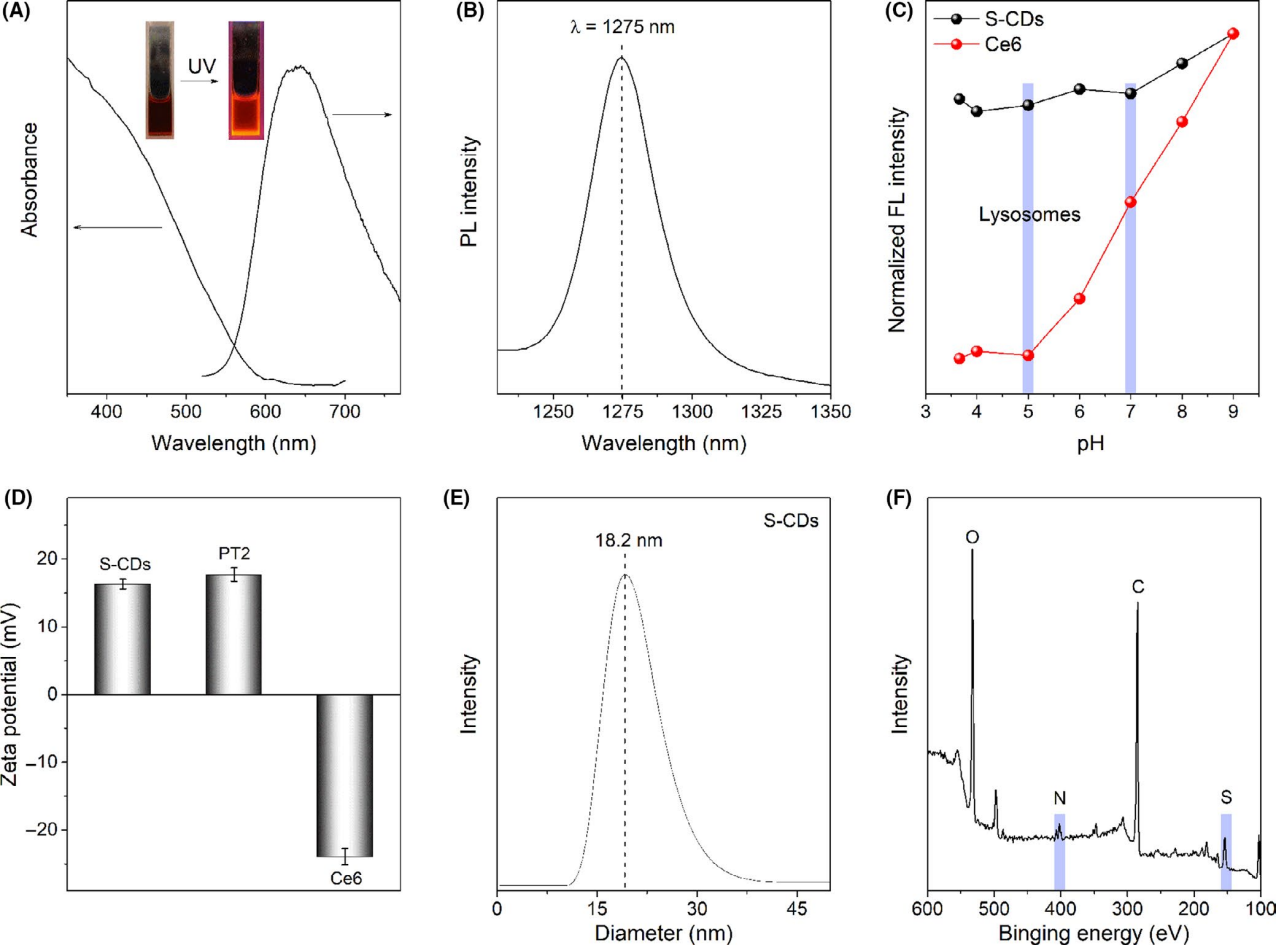
Characterization of S‐CDs: A, UV − vis absorption and fluorescence emission spectra (λex = 420 nm); B, Characterized ^1^O_2_ phosphorescence emissions at 1275 nm (CH_3_CN‐D_2_O mixture solvent, 15:1, V/V); C, Fluorescence intensity of the S‐CDs, Ce6 and PT2 in the pH range from 3.7 to 9; D, Zeta potential of S‐CDs, Ce6 and PT2; E, Dynamic light scattering; and F, XPS survey spectrum of the S‐CDs

S‐CDs was prepared via a hydrothermal synthetic route from a polythiophene precursor (PT2) (Figure S1A). As shown in Figure 1A, The UV‐vis absorption of the as‐prepared S‐CDs spans from 400 to 700 nm, with red fluorescence emission at ~650 nm (λex = 420 nm), which are similar to those reported previously.^8^ The characteristic phosphorescence of ^1^O_2_ at 1275 nm was observed from S‐CDs upon excitation at 420 nm (Figure 1B). Using Rose Bengal as the standard (Φ_Δ_ = 0.75),^19^ singlet oxygen quantum yield (QY) of ∼0.95 was determined, and for classic PS, Ce6, the ^1^O_2_ QY was detected as ∼0.27, much lower than that of S‐CDs. Furthermore, the fluorescence of S‐CDs was almost unchanged upon lowering the pH from 7 to 5, while that of Ce6 was severely reduced (Figure 1C). Since cells usually possess neutral environment with the lowest pH in lysosomes (~5), the pH stability of S‐CDs ensures it an excellent photosensitizer for photodynamic therapy of cancer cells. Meanwhile, the surface of S‐CDs was positively charged (16 mV, Figure 1D), which is favourable for its uptake by cells. Besides, the size of S‐CDs determined with transmission electron microscopy (TEM) (Figure S2) was ranging from 10 to 30 nm, consistent with hydrodynamic size of S‐CDs (Figure 1E). High‐resolution TEM (HRTEM) revealed the crystallinity of the S‐CDs with the interplanar distance of 0.205 nm (Figure S2). Moreover, sulphur doping was confirmed with X‐ray photoelectron spectroscopy. As illustrated in Figure 1F, besides C and O, S and N were presented in this particle. These results indicated the successful synthesis of S‐CDs, and display the excellent properties of the nano‐PS.

### 3.2 In vitro PDT efficiency

**Figure 2:**
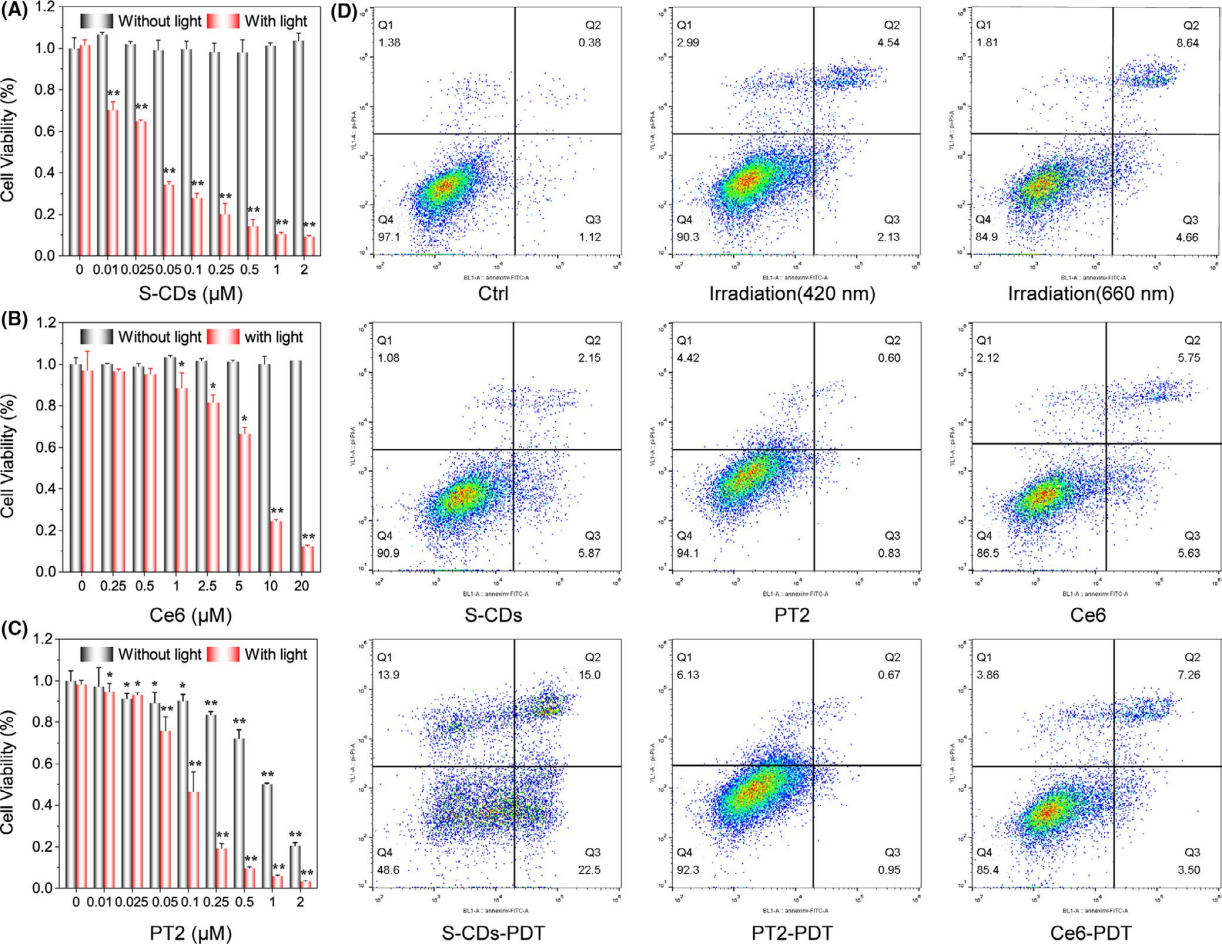
Comparison of the PDT performance of S‐CDs with Ce6 and PT2 with U87‐MG cells: A, S‐CDs in the concentration range 0.01‐2 µmol/L. Data are presented as mean ± SD (n = 3). Statistical analysis: *P < .05. **P < .01; B, Ce6 in the concentration range 0.01‐2 µmol/L. Data are presented as mean ± SD (n = 3). Statistical analysis: *P < .05. **P < .01; C, PT2 in the concentration range 0.25‐20 µmol/L. Data are presented as mean ± SD (n = 3). Statistical analysis: *P < .05. **P < .01; D, Cell apoptosis analysis of S‐CDs, Ce6 and PT2 mediated PDT by Annexin V‐FITC/PI staining and subsequent flow cytometry assay

The uptake of S‐CDs by U87‐MG cells was characterized with confocal laser scanning microscopy and flow cytometry (Figure S3). After incubation with 0.25 µmol/L S‐CDs for 12 hours, about 89.8% of the U87‐MG cells could uptake S‐CDs, and the particles were located mainly in cytoplasm. The cytotoxicity and PDT efficiency of S‐CDs against U87‐MG were evaluated by CCK‐8 assay. As illustrated in Figure 2A, S‐CDs have little effect on the survival of U87‐MG cells in the dark even at 2 µmol/L, in contrast, about 30% cell death was observed in 0.01 µmol/L S‐CDs mediated PDT. Besides, the survival rate of 10% was obtained when increasing the S‐CDs concentration to 1 µmol/L. For the same concentration of Ce6 and PT2, much less cell death was observed (Figure 2B,C), indicating the high photo‐oxidative activity of S‐CDs. Besides, after PDT with S‐CDs, the morphology of the cells changes greatly as blebbing, shrinkage and nuclear fragmentation (Figure S4), which is coincide with the morphology changes of cell apoptosis. To further confirm the way of cell death, Annexin V‐FITC/PI was employed. As shown in Figure 2D, neither Annexin V nor PI‐stained cells were detected before PDT. After S‐CDs mediated PDT, the percentage of Annexin V positive cells was visibly increased, indicating that apoptosis was the major way of cell death. Moreover, neither Annexin V nor PI positive cells were observed when cells were treated with S‐CDs or irradiation only, also confirming the excellent biocompatibility of S‐CDs. All of the results indicated the high efficiency and pretty biocompatibility, besides the investigation of positive PS suggested the good PDT performance of S‐CDs should be ascribed to its high photo‐oxidative activity, but not the positive charge.

### 3.3 Regulation of signalling pathways in S‐CDs mediated PDT

**Figure 3:**
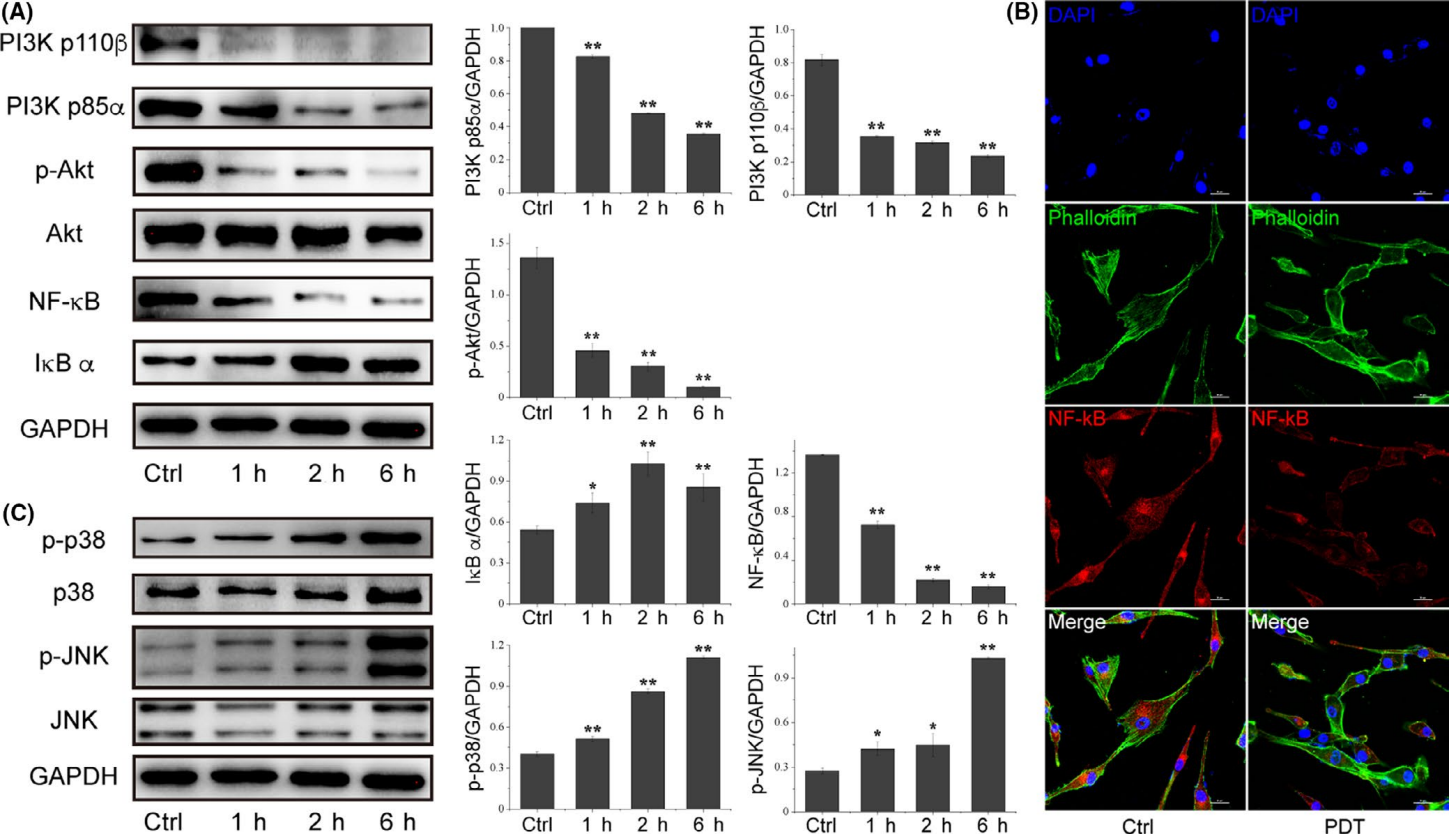
PI3K/Akt pathway variated after S‐CDs mediated PDT. A, Western blot images and quantitative analysis of the change of PI3K/Akt/NF‐κB signalling proteins. GAPDH was used as an internal control. Data are presented as mean ± SD (n = 3). Statistical analysis: *P < .05. **P < .01. B, Immunofluorescence images of NF‐κB after S‐CDs mediated PDT. C, Western blot images and quantitative analysis of the expression of p38/JNK signalling pathway proteins. GAPDH was used as an internal control. Data are presented as mean ± SD (n = 3). Statistical analysis: *P < .05. **P < .01

To dissect the superior PDT performance of S‐CDs in killing cancer cells, and also confirm if S‐CDs could function as a PI3K/Akt inhibitor, PI3K/Akt signalling pathway was detected here. As shown in Figure 3A, downregulation of PI3K p85α, PI3K p110β and p‐Akt were obviously observed, indicating the inhibition of the PI3K/Akt pathway by S‐CDs mediated PDT. Quantitative analysis showed significant suppression of the p‐Akt at 1 hour after PDT, and the level continued to decline over time. In contrast, Ce6 and PT2 with the same concentration plus irradiation could not induce the inhibition of PI3K/Akt pathway (Figure S5). In our study, the downstream NF‐κB signalling pathway was inhibited through upregulation of IκB and downregulation and transferred localization of NF‐κB was validated, confirming the inactivation of NF‐κB (Figure 3A,B). Thus, cell survival could be inhibited followed by the inhibition of PI3K/Akt pathway in S‐CDs mediated PDT. Herein, variation of p38 and JNK, cell apoptosis‐related pathway, after S‐CDs mediated PDT was also analysed by Western blot. As shown in Figure 3C, significantly enhanced p‐p38 at 1 hour after PDT was observed, while p‐JNK showed slight increment and last to 6 hours. For the same concentrations of Ce6 and PT2, no appreciable variation of p‐p38 and p‐JNK was observed at 6 hours after treatment (Figure S5).

### 3.4 Variation of apoptosis proteins after S‐CDs mediated PDT

**Figure 4:**
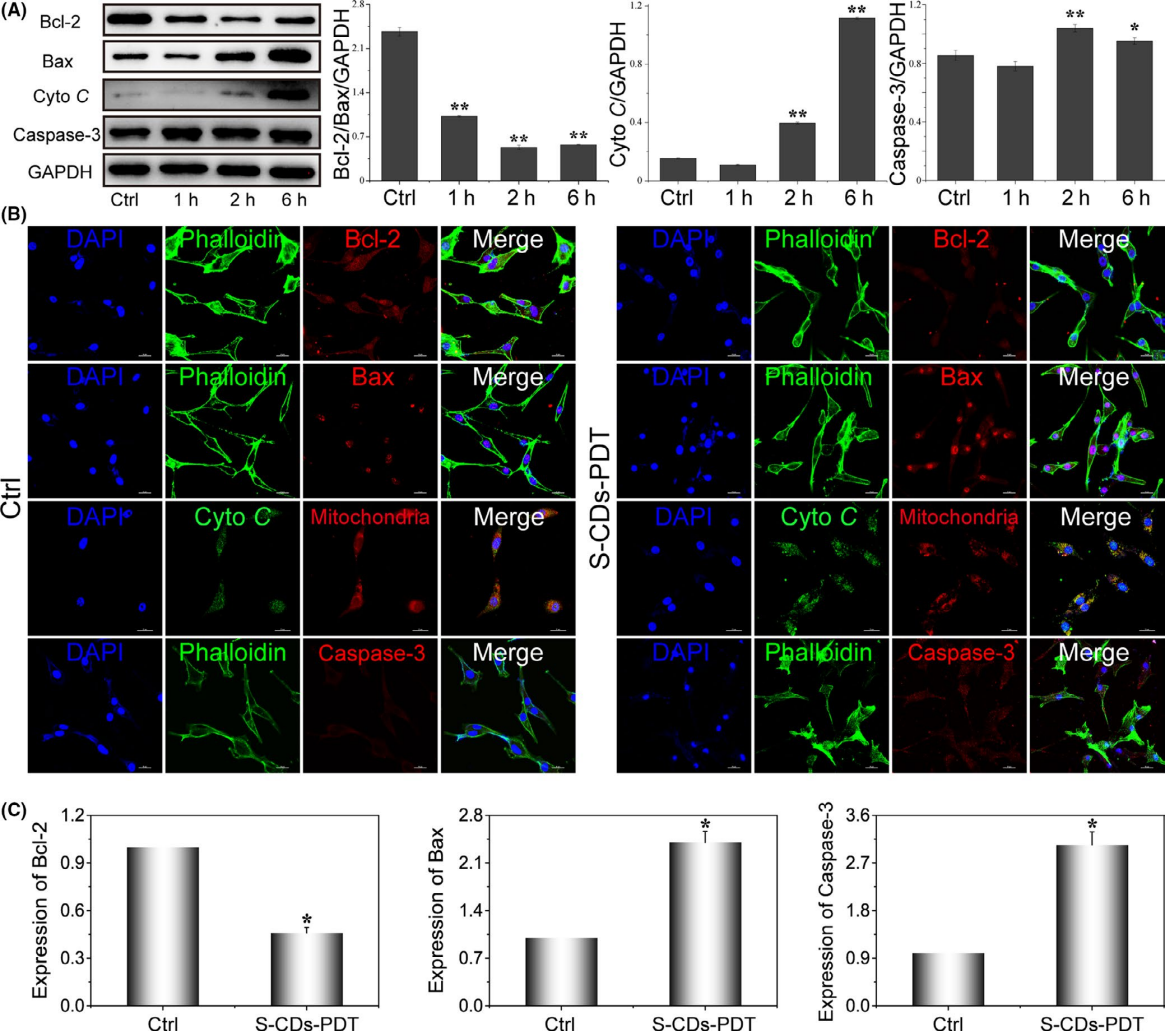
Investigations on the expression of proteins and genes related to cell apoptosis. A, Western blot images and quantitative analysis of apoptosis proteins after PDT mediated by S‐CDs, samples were probed with anti‐GAPDH, anti‐Bcl‐2, anti‐Bax, anti‐Cyto *C* and anti‐caspase3. GAPDH was used as an internal control. Data are presented as mean ± SD (n = 3). Statistical analysis: *P < .05. **P < .01. B, The immunofluorescence images showing the expression of the essential proteins closely related to apoptosis (Bcl‐2, Bax, Cyto *C* and caspase3) after S‐CDs mediated PDT. Scale bar: 20 μm. C, Quantitative real‐time PCR analysis of the expression of cell apoptosis‐related genes in U87‐MG after PDT. Data are presented as mean ± SD (n = 3). Statistical analysis: *P < .05. **P < .01

The expression of apoptosis proteins and the detailed intracellular responses to PDT mediated by S‐CDs were further discussed. As illustrated in Figure 3C, the Bcl‐2/Bax ratio was significantly decreased after S‐CDs mediated PDT and Cyto *C* showed a great release (Figure 4A). The variation of mRNA level of Bcl‐2 and Bax was also detected. The transcription levels of Bcl‐2 was decreased and Bax was increased, which was consistence with the protein levels (Figure 4C). However, control investigations with Ce6 and PT2 at the same concentration plus irradiation induced minimal activation of the mitochondria apoptosis pathway (Figure S6), also indicating the high efficiency of the nano‐PS. The imbalance of Bcl‐2 family proteins could result in the collapse of mitochondria and release of Cyto *C*. Western blot and immunofluorescence results demonstrated the release of Cyto *C* from mitochondria (Figure 4A,B) and the images also displayed the collapse of mitochondria with the ambiguous border of mitochondira. Then, the expression of Caspase‐3 in U87‐MG cells after PDT was further examined. As shown in Figure 4A,B, boosted Caspase‐3 was observed after S‐CDs mediated PDT. These results demonstrated that S‐CDs induced cell apoptosis through mitochondria pathway with higher efficiency over the classical organic photosensitizers.

### 3.5 The role of ROS during S‐CDs mediated PDT

**Figure 5:**
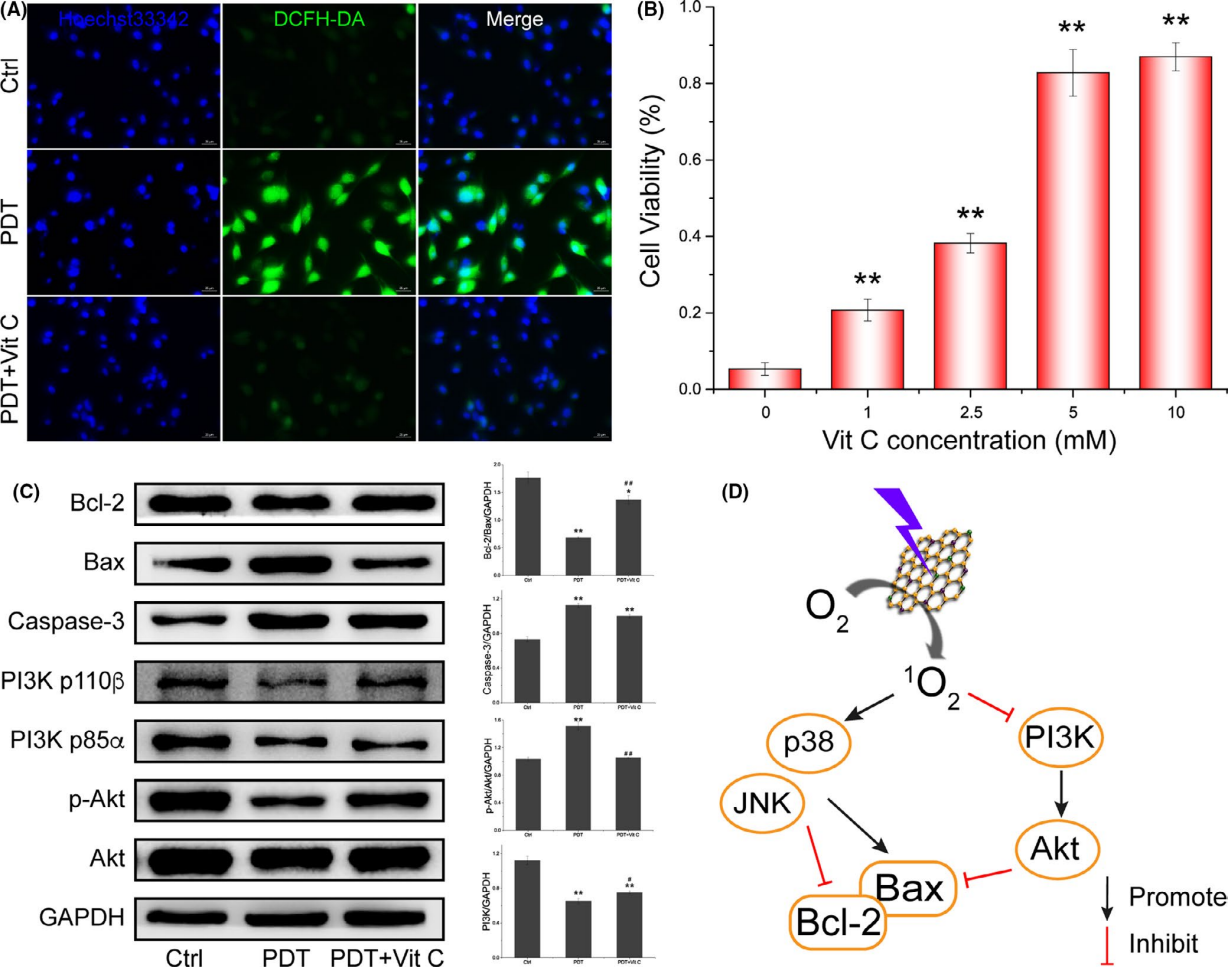
Detection of ROS and pro‐apoptotic factors in U87‐MG. A, Fluorescent images of ROS generated during S‐CDs‐mediated PDT with a ROS probe, DCFH‐DA. B, Cell viability of U87‐MG after S‐CDs mediated PDT with or without the presence of Vitamin C. C, Western blot images and quantitative analysis of the change of apoptosis‐related proteins and signalling pathway. GAPDH was used as an internal control. Data are presented as mean ± SD (n = 3). Statistical analysis: *P < .05. **P < .01. D, Scheme of signalling pathway changes after S‐CDs mediated PDT

S‐CDs possessed high singlet oxygen QY, therefore, to elucidate the ROS generation in cells, cells were stained with a ROS probe (DCFH‐DA). Through fluorescent inverted microscopy test, obvious production of ROS in U87‐MG (Figure 5A) was identified after PDT, while the cells with S‐CDs or irradiation only did not show bright fluorescence (Figure S7). After 12 hours incubation, most particles were uptaken by cancer cells and located in mitochondria and lysosome parallelly (Figure S8). Therefore, the simultaneous occurrence of S‐CDs in mitochondria and lysosome may also contribute to the high PDT efficiency of S‐CDs. The release of Cyto *C* has been demonstrated in Figure 4A,B and as evidenced from the enhanced green fluorescence from Fluo‐4 AM (a Ca^2+^ probe), obviously enhanced cytoplasmic free calcium was observed in PDT group over the control group (Figure S9). Therefore, the above data suggested that high yield ROS was generated in cells upon S‐CDs mediated PDT, resulting in enhanced MOMP, which promote the release of apoptotic factors such as Cyto *C* and free calcium, and finally initiate the cell apoptosis.

Moreover, vitamin C was explored as a ROS scavenger to verify the effect of ROS in cell apoptosis. The presence of vitamin C in the cells led to clearance of ROS generated by S‐CDs mediated photosensitization (Figure 5A). To further verify the effect of ROS on PI3K/Akt signalling pathways, Western blot was employed to detect the change of PI3K/Akt pathway. As illustrated in Figure 5B, the cell death was significantly reversed after different concentration of Vit C treatment. Moreover, the change of mitochondria pathway proteins and the inhibition of PI3K/Akt signalling pathway could be reserved after Vit C application (Figure 5C,D), also indicating that ROS generated in cancer cells played a vital roles in the S‐CDs‐PDT mediated killing process. Above all, S‐CDs mediated PDT validly inhibited PI3K/Akt signalling pathway and activated p38/JNK signalling pathway, and the changed pathways then suppress Bcl‐2 and boost Bax, leading to the collapse of mitochondria and eventually cell apoptosis.

### 3.6 PI3K/Akt inhibition in malignant melanoma cell

**Figure 6:**
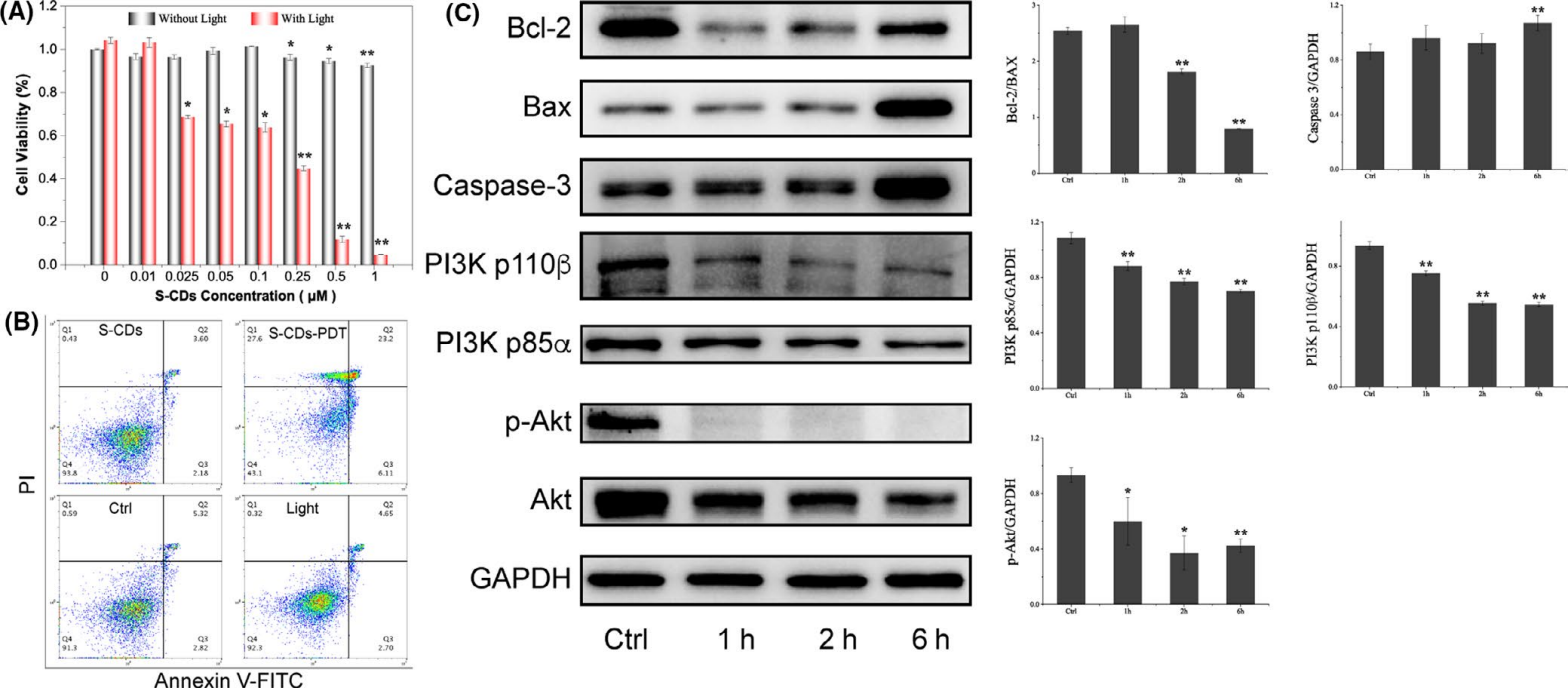
A375 response to S‐CDs mediated PDT. A, Cell viability of A375 after S‐CDs mediated PDT in the concentration range 0.01‐1 µmol/L; B, Cell apoptosis analysis of S‐CDs mediated PDT by Annexin V‐FITC/PI staining and subsequent flow cytometry assay; C, Western blot images and quantitative analysis of the apoptotic proteins (Bcl‐2, Bax and caspase‐3) and PI3K/Akt signalling proteins after S‐CDs mediated PDT. Data are presented as mean ± SD (n = 3). Statistical analysis: *P < .05. **P < .01

To further demonstrate the inhibition of PI3K/Akt signalling pathway, we choose another cancer cell, malignant melanoma cell line A375, to evaluate the biological effect of S‐CDs. As illustrated in Figure 6A, low concentration of S‐CDs mediated PDT could induce most A375 cell death, indicating the high efficiency of S‐CDs. Annexin V/PI staining results showed that similar to U87‐MG, A375 died mainly through cell apoptosis (Figure 6B). Furthermore, the mechanism of S‐CDs mediated PDT in A375 was also detected. Western blot images and quantitative analysis showed that the level of Bcl‐2 protein was significantly decreased after S‐CDs mediated PDT, and Bax showed increased expression (Figure 6C). Importantly, variation of PI3K/Akt signalling pathway during S‐CDs mediated PDT in A375 was coincide with U87‐MG, and the activated PI3K/Akt was significantly inhibited after PDT (Figure 6C). On the basis of the above analysis, we speculate that S‐CDs could act as an inhibitor of the aberrantly activated PI3K/Akt signalling pathway against cancer cells.

## 4 DISCUSSION

In the race for wide application of PDT, PSs majorly experienced fourth generations of developments.^20^, ^21^, ^22^ Due to the unique quantum confinement effect of nanomaterials, excellent optical characteristics, such as tunable excitation and emission wavelength and low photo‐bleaching, are witnessed in nanoscale PSs. Besides, the toxicity and biocompatibility of nano‐PSs can also be adjusted. Therefore, nano‐PSs are denoted as the fourth generation of PSs.^21^ Compared with conventional organic PSs, nano‐PSs are suitable for simultaneous tumour fluorescence imaging and PDT (image‐guided PDT).^14^ A variety of nano‐PSs, such as carbon dots,^8^, ^23^ nanoscale metal‐organic frameworks,^21^ porous silicon^24^ and two dimensional materials,^25^, ^26^ have been explored for the diagnosis and therapy of cancer. Herein, S‐CDs possessed small size and positive charge, which was beneficial for cells uptake. Compared with the classic organic PS, S‐CDs showed higher singlet oxygen quantum yield of 0.95 and better stability (Table S3).

During PDT, reactive oxygen species (ROS), especially singlet oxygen (^1^O_2_), generated by photosensitization ultimately induces cell death and tumour elimination.^10^, ^27^, ^28^ It was found that mitochondria and lysosome were the most popular location and accumulation sites for photosensitizers, and PDT‐mediated cell apoptosis occurred when the photosensitizers located mainly in mitochondria or lysosome.^29^ ROS generated in these organelles subsequently induced mitochondrial outer membrane permeabilization (MOMP), releasing the apoptosis‐related factors such as cyto *C* and Ca^2+^ from internal stores to cytoplasm,^30^, ^31^ which triggered the caspase cascade,^12^ and finally mitochondria mediated cell apoptosis.^9^, ^32^ Furthermore, parallel localization of PSs in both mitochondria and lysosome can greatly improve the PDT efficiency and cell death rate.^33^ In this study, S‐CDs could actively enter cancer cells, and located in both mitochondria and lysosome. The highly uptake efficiency may be ascribed to the small positive surface charge of S‐CDs, and the parallel localization in both mitochondria and lysosome may result in the high efficiency of S‐CDs.

Mitochondria mediated cell apoptosis was reported the most majority mode of cell death in PDT and was resultant from the change of the Bcl‐2 family proteins with the change of several signalling pathways. Among these, p38 and JNK, members of MAPKs, which closely related to the induction of apoptosis,^34^ can directly regulate the translocation of Bax and the process of anti‐apoptotic proteins phosphorylation respectively.^35^, ^36^ The activation of p38 and JNK has been frequently observed in response to PDT, followed by inducing phosphorylation of Bcl‐2 family proteins and elicits MOMP.^37^, ^38^, ^39^ PI3K/Akt is one of the most frequently activated signalling networks during tumorigenesis. Upon activation by PI3K, Akt is phosphorylated and then inhibits the function of the pro‐apoptotic Bcl‐2 family members, resulting in cell death inhibition.^13^, ^40^ NF‐κB is a transcription factor and also a downstream effector of the Akt pathway,^41^ and it could inhibit early apoptosis of glioma cells by promoting the expression of Bcl‐2.^34^, ^42^ The variation of these signalling pathways resulted in imbalance of the Bcl‐2 family proteins, including decreased anti‐apoptotic proteins (such as Bcl‐2) and increased pro‐apoptotic proteins (such as Bax).^43^, ^44^ On the basis of our results, we revealed that S‐CDs mediated PDT could promote the apoptosis‐related pathway, p38/JNK, which accelerate cancer cell apoptosis, and simultaneously inhibited PI3K/Akt signalling even at a low concentration, which decline the survival ability of cancer cells.

In conclusion, we for the first time suggested that sulphur doped carbon dots (S‐CDs) used as nano‐PS could instigate potent cancer cell apoptosis as a PI3K/Akt inhibitor. Compared with the previous organic photosensitizers, S‐CDs possess higher singlet oxygen quantum yield of 0.95. Upon cell uptake, S‐CDs located mainly in mitochondria and lysosome, and during PDT, photosensitization validly inhibited PI3K/Akt pathway and activated p38/JNK pathway. Both of the changes could suppress the anti‐apoptotic Bcl‐2 members and boost the pro‐apoptotic Bcl‐2 members, leading to the collapse of mitochondria, release of apoptosis factors, caspase cascades and eventually resulted in cell apoptosis. Considering the ongoing efforts in highly efficient nano‐PSs developments, it is expected that more signalling pathways will be perturbed, leading to higher cell death rates. This could move us closer to understanding the mechanism of nano‐PS mediated PDT and provide vital information for drug development.

## CONFLICTS OF INTEREST

There are no conflicts to declare.

## AUTHORS’ CONTRIBUTIONS

Yanjing Li and Ronghui Zhou: collection and/or assembly of data and manuscript writing; Shihong Wu: synthesis of the nano‐PS; Junjiang Zhang: figure processing; Xiaoxiao Cai: conception and design, data analysis and interpretation, financial support and final approval of manuscript.

## Supporting information

Supplementary Material

## Data Availability

The data that support the findings of this study are available from the corresponding author upon reasonable request.
