# Supplementary material for: Sulphur doped carbon dots enhance photodynamic therapy via PI3K/Akt signalling pathway
Source: Cell Prolif. 2020 May 4;53(5):e12821. doi: 10.1111/cpr.12821 (PMC7260068; doi:10.1111/cpr.12821)
Supplement: Supplementary file 1 — Supplementary Material [file CPR-53-e12821-s001.docx]

**Sulfur Doped Carbon Dots enhance photodynamic therapy via PI3K/Akt signaling pathway**

**Running title: Sulfur Doped Carbon Dots enhance PDT**

Yanjing Li^1^,Shihong Wu^2^, Junjiang Zhang^3^, Ronghui Zhou^1,^ *, and Xiaoxiao Cai^1,^ *

Yanjing Li: Master's degree; Shihong Wu: Bachelor's degree; Junjiang Zhang: Master's degree; Ronghui Zhou: Ph.D., Xiaoxiao Cai: Ph.D.

^1^ State Key Laboratory of Oral Diseases, West China Hospital of Stomatology, Sichuan University, Chengdu 610041, China

^2^ Analytical & Testing Center, Sichuan University, Chengdu 610064, China

^3^ Department of Prosthodontics, Tianjin Medical University, Tianjin 300203, China

***Corresponding authors:**

Ronghui Zhou

E-mail address: zhouronghui@scu.edu.cn

Xiaoxiao Cai

E-mail address: [xcai@scu.edu.cn](mailto:xcai@scu.edu.cn)

**Supplementary Figure Legends**

**Supplementary Figure Legends**

**Figure S1.** Schematic of the route for synthesis of S-CDs.


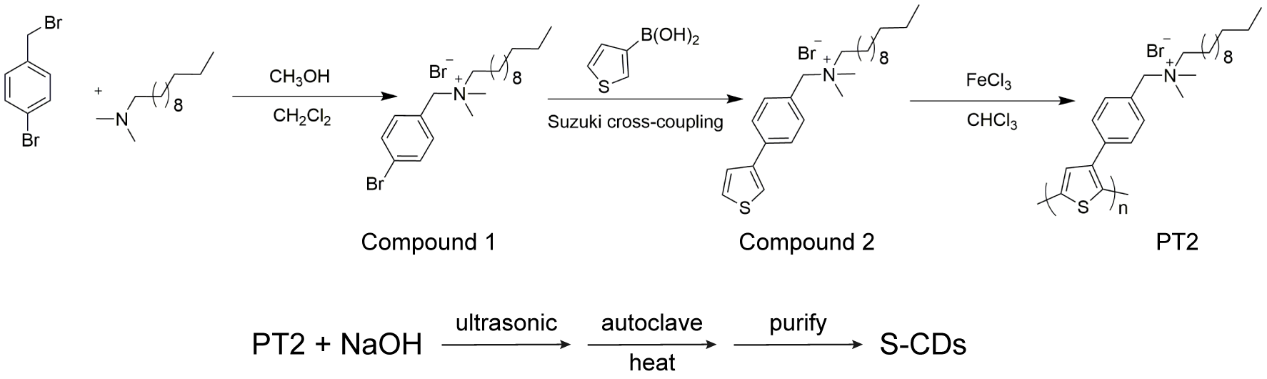


**Figure S2.** TEM image and HR-TEM images of S-CDs.


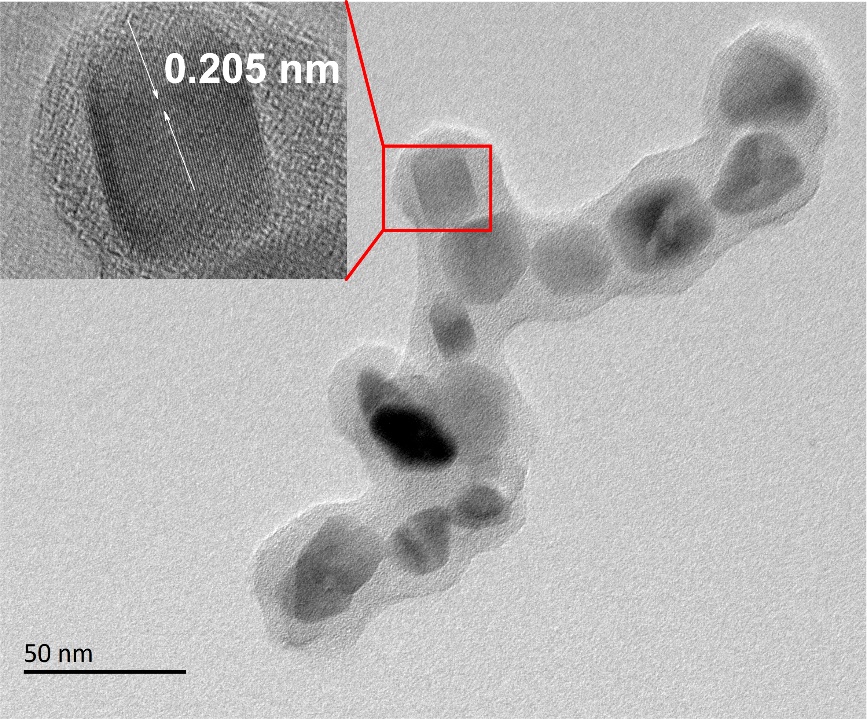


**Figure S3.** The uptake of S-CDs by U87-MG was analyzed by confocal laser scanning microscope and flow cytometry. Scale bars: 25 μm.


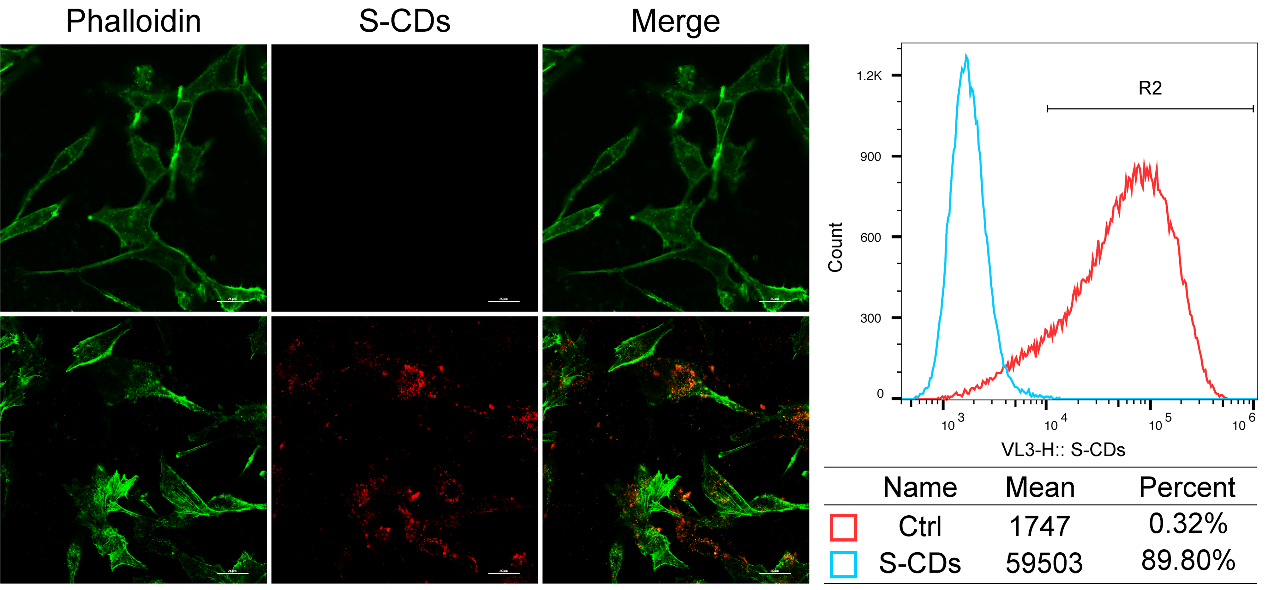


**Figure S4.** Bright field of U87-MG after S-CDs mediated PDT. Scale bars: 25 μm.


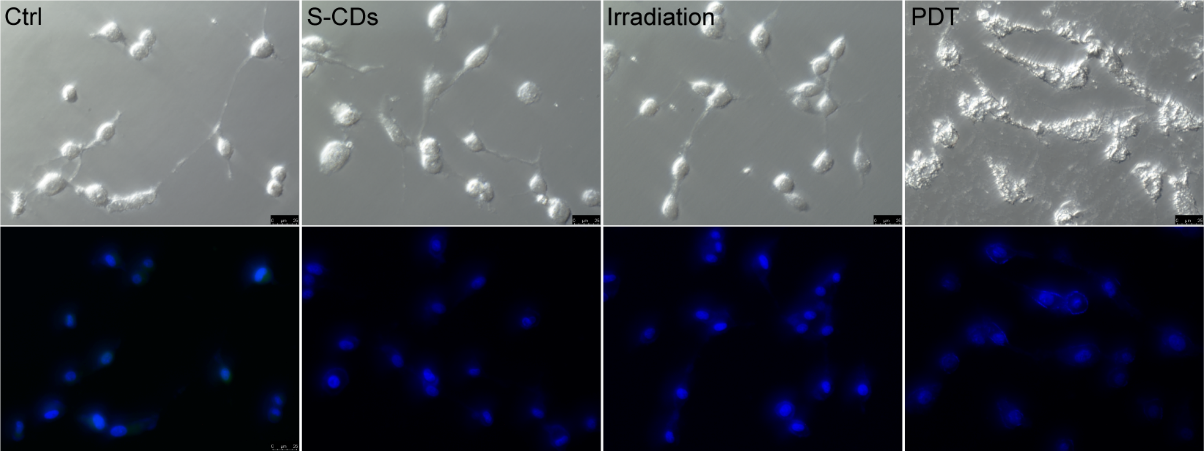


**Figure S5.** Western blot images of the expression of PI3K/Akt and p38/JNK signaling pathway proteins in Ce6 and PT2 mediated PDT. GAPDH was used as an internal control.


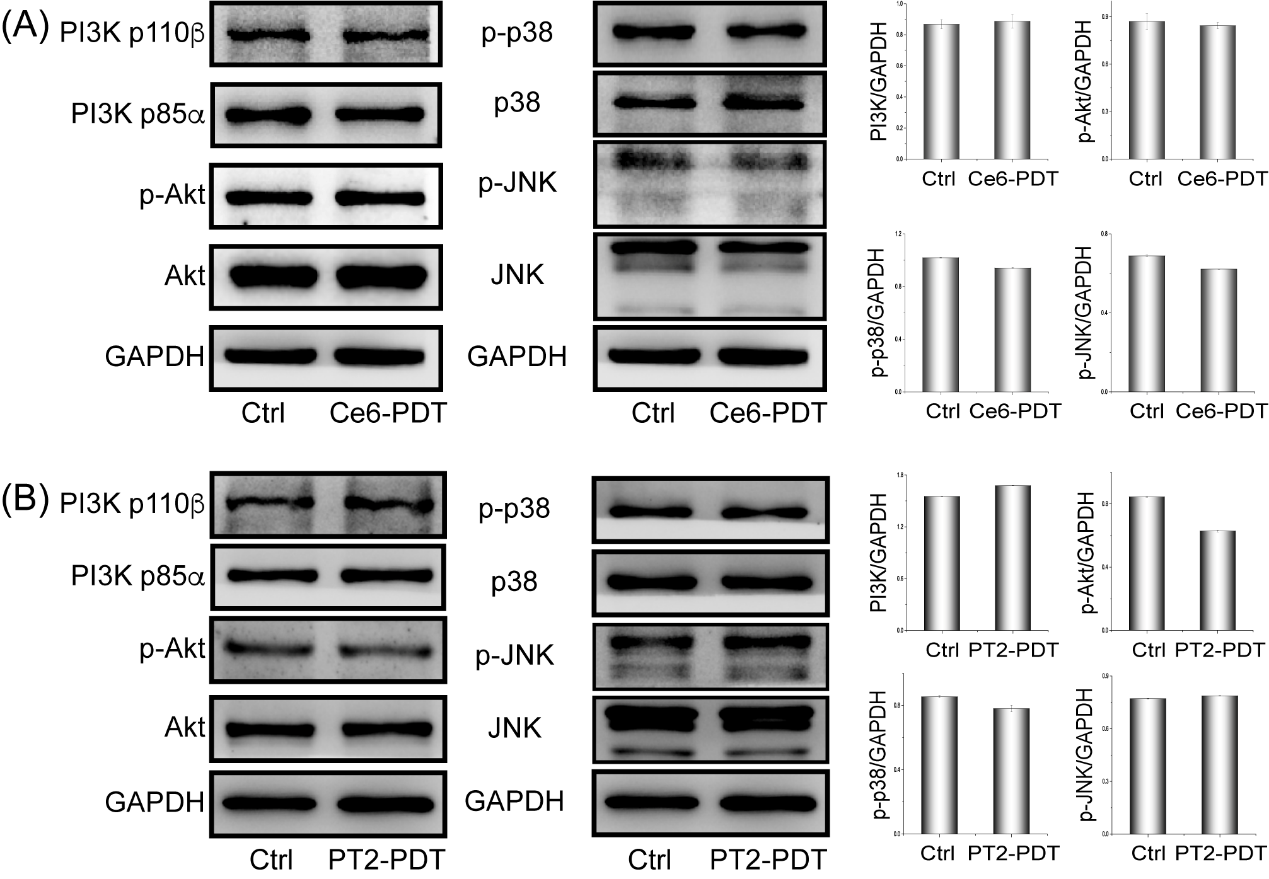


**Figure S6.** Western blot images of the expression of cell apoptosis proteins in Ce6 and PT2 mediated PDT. GAPDH was used as an internal control.


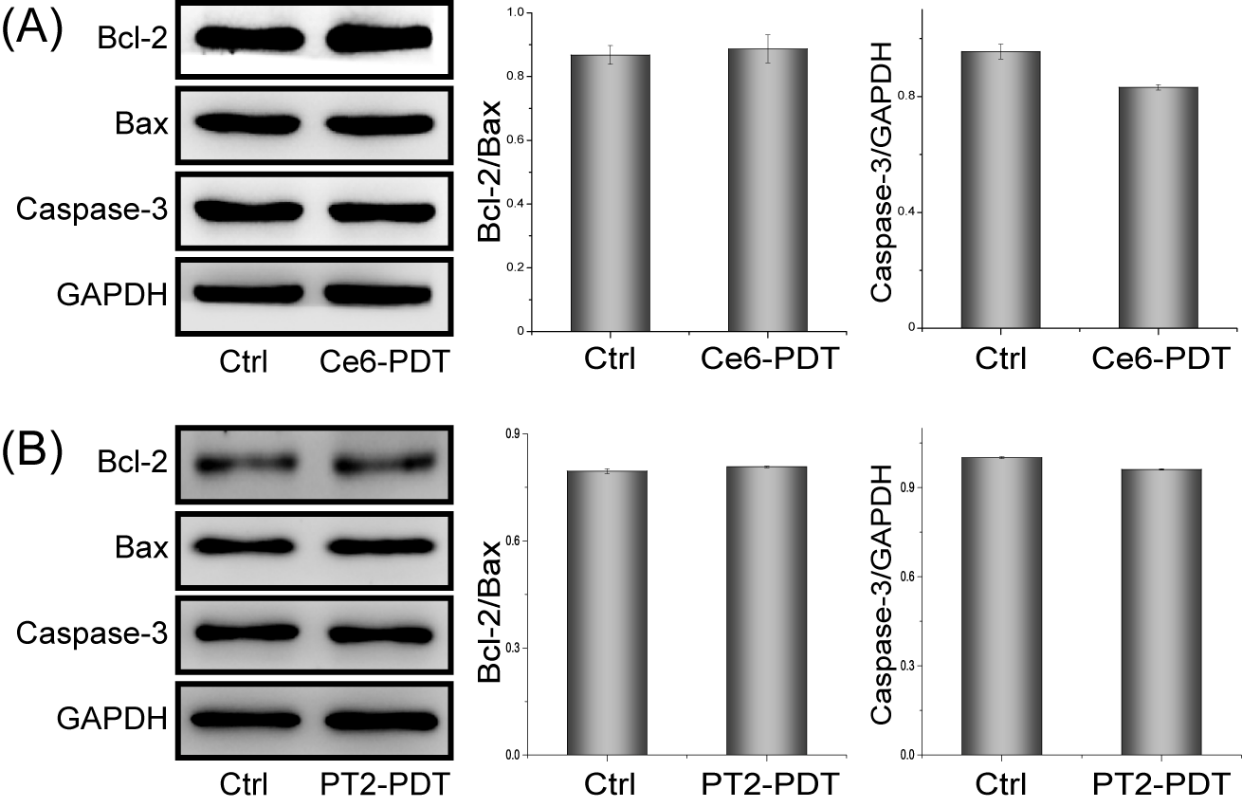


**Figure S7.** Detection of ROS in U87-MG. Corresponding fluorescence images of ROS in U87-MG after S-CDs incubation and irradiation only. Scale bars: 25 μm.


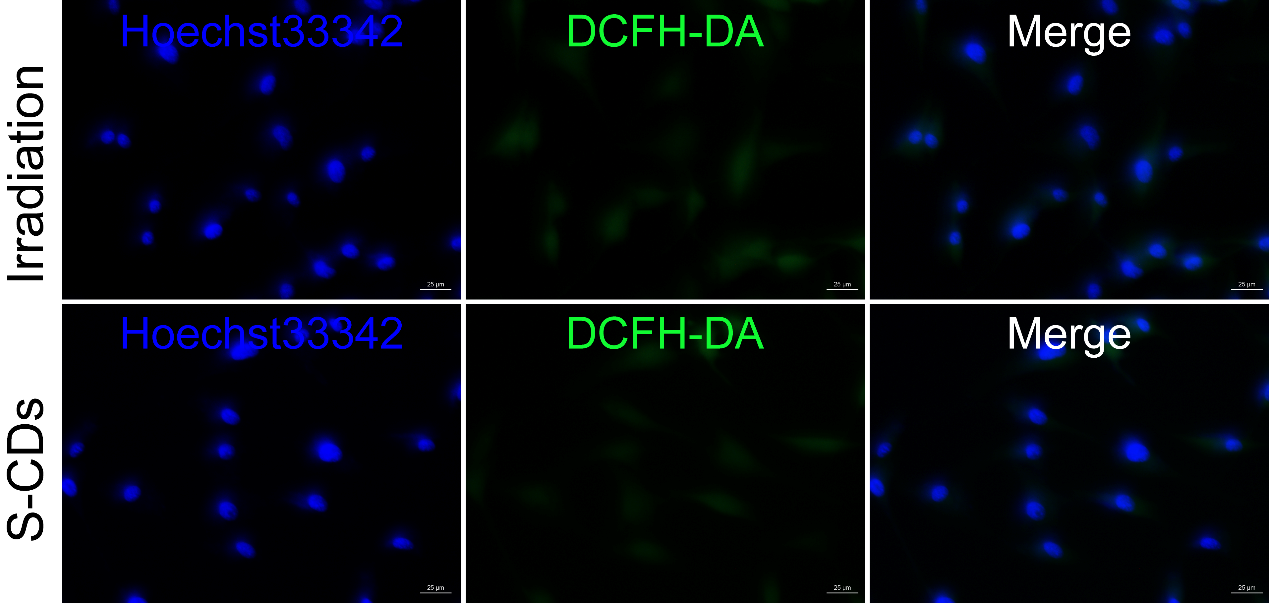


**Figure S8.** Subcellular localization of S-CDs in U87-MG cells: (A) fluorescence images of U87-MG cells incubated with S-CDs (0.5 µM) and Mito-tracker green (20 nM); and (B) fluorescence images of U87-MG cells incubated with S-CDs (0.5 µM) and Lyso-tracker green (50 nM). Scale bar: 25 μm.


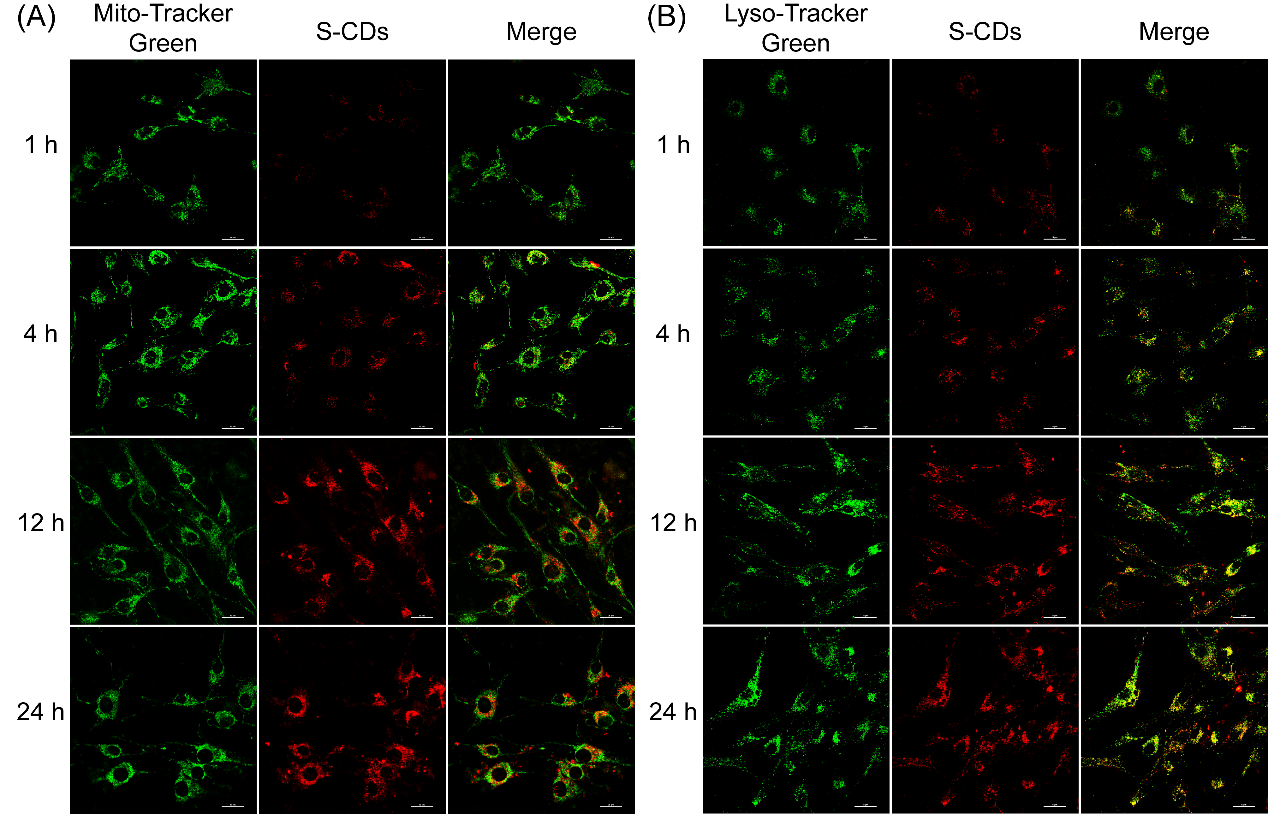


**Figure S9.** Fluorescence images of free calcium in U87-MG after S-CDs mediated PDT. Scale bars: 25 μm.


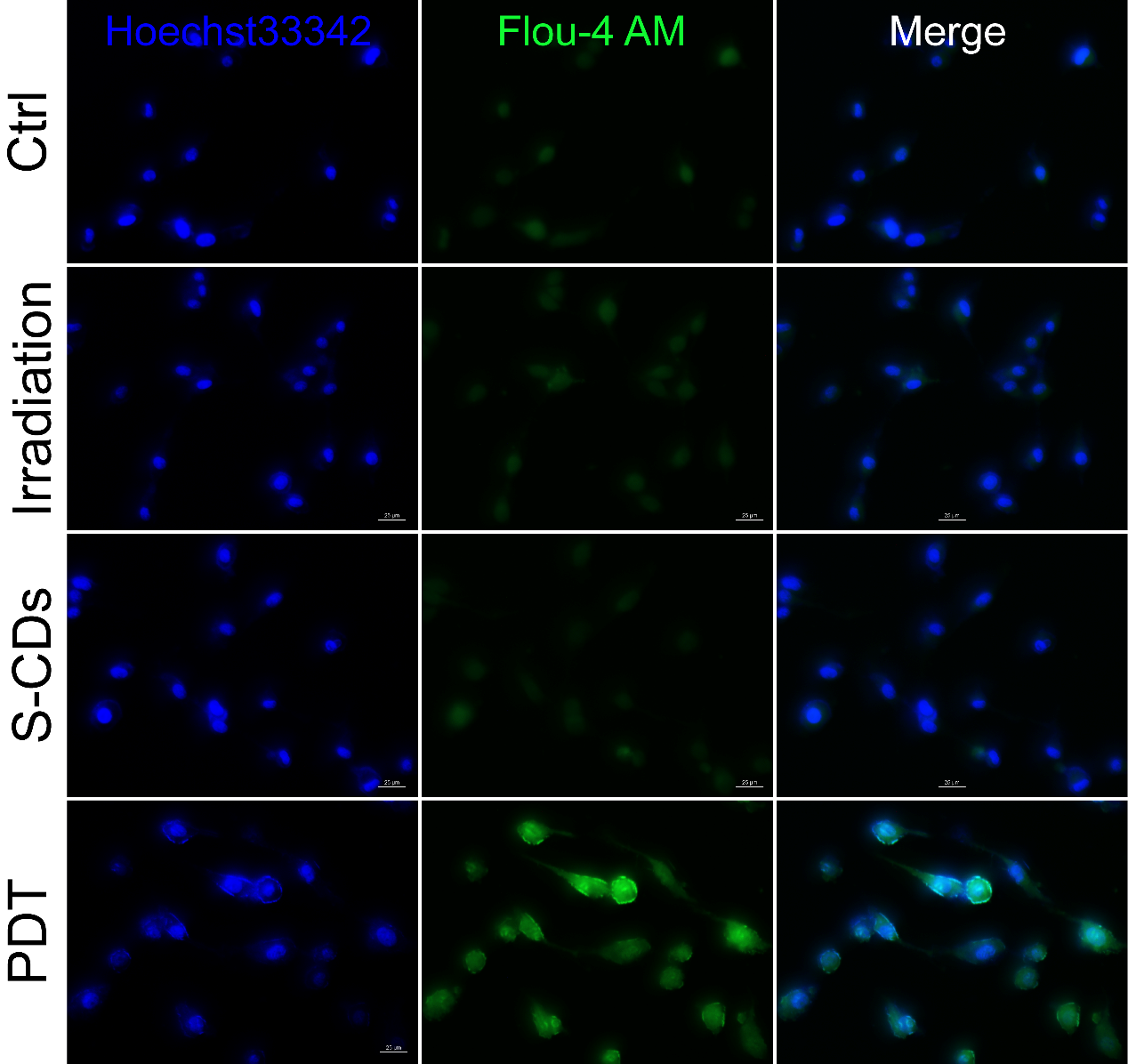


**Supplementary Tables**

**Table S1**. The instrumental information used for characterizations in this work.

| Characterization items | Instrumet model | Manufacturer |
| --- | --- | --- |
| Absorption spectra | UV-1750 | Shimadzu, Japan |
| Absorption spectra | Lamda365 | Perkin-Elmer, USA |
| Diffused reflectance spectra | UV-3600 | Shimadzu, Japan |
| TEM | Tecnai G2 F20 S-TWIN  Acceleration voltage: 200 kV | FEI, USA |
| XPS | AXIS Ultra DLD  Excitation: Al-Kα X-ray | Kratos, Japan |
| DLS&Zeta potential | Zetasizer Nano ZS | Malvern, England |
| CLSM | AIR-MP | Nikon, Japan |
| Flow Cytometry | Attune NxT | Life, USA |

**Table S2.** Primers of target genes.

| Primer | Sequence |
| --- | --- |
| GAPDH-F | CAGGAGGCATTGCTGATGAT |
| GAPDH-R | GAAGGCTGGGGCTCATTT |
| Bcl-2-F | AACATCGCCCTGTGGATGAC |
| Bcl-2-R | GACTTCACTTGTGGCCCAGAT |
| Bax-F | TCATGGGCTGGACATTGGAC |
| Bax-R | GAGACAGGGACATCAGTCGC |
| CPP32-F | CCAAAGATCATACATGGAAGGG |
| CPP32-R | CTGAATGTTTCCCTGAGGTTTG |

**Table S3**. Comparison of S-CDs, Ce6, and PT2, and low concentration of PSs mediated PDT.

| PS | Signaling Pathways | | Physicochemical property | | |
| --- | --- | --- | --- | --- | --- |
|  | PI3K/Akt | p38/JNK | ^1^O_2_ QY | Charge | Stability |
| S-CDs | ↓ | ↑ | ~0.95 | + | Good |
| Ce6 | — | — | <0.5 | - | Poor |
| PT2 | — | — | <0.5 | + | Poor |
